# Different Susceptibilities between *Apoe*- and *Ldlr*-Deficient Mice to Inflammation-Associated Colorectal Carcinogenesis

**DOI:** 10.3390/ijms17111806

**Published:** 2016-10-28

**Authors:** Takuji Tanaka, Takeru Oyama, Shigeyuki Sugie, Masahito Shimizu

**Affiliations:** 1 Department of Diagnostic Pathology (DDP) and Research Center of Diagnostic Pathology (RC-DiP), Gifu Municipal Hospital, 7-1 Kashima-cho, Gifu City, Gifu 500-8513, Japan; 2 Department of Tumor Pathology, Gifu University Graduate School of Medicine, 1-1 Yanagido, Gifu City, Gifu 501-1194, Japan; 3 Department of Molecular and Cellular Pathology, Graduate School of Medical Science, Kanazawa University, Kanazawa, Ishikawa 920-8640, Japan; takeruoyama@staff.kanazawa-u.ac.jp; 4 Department of Pathology, Murakami Memorial Hospital, Asahi University, School of Dentistry, 3-23 Hashimoto-cho, Gifu City, Gifu 500-8523, Japan; sugie@murakami.asahi-u.ac.jp; 5 Department of Gastroenterology/Internal Medicine, Gifu University Graduate School of Medicine, 1-1 Yanagido, Gifu City, Gifu 501-1194, Japan; shimim-gif@umin.ac.jp

**Keywords:** *Apoe*, *Ldl* receptor, genetically altered mice, serum lipid profiles, inflammation, colorectal carcinogenesis

## Abstract

Hypercholesterolemia resulting in atherosclerosis is associated with an increased risk of ischemic heart disease and colorectal cancer (CRC). However, the roles of apoliprotein (Apo) E (*Apoe*) and low-density lipoprotein (*Ldl*) receptor (*Ldlr*) in colorectal carcinogenesis have not yet been investigated. In this study, we examined the susceptibility of *Apoe*-deficient and *Ldlr*-deficient mice, which are genetic animal models of atherosclerosis to azoxymethane (AOM)/dextran sodium sulfate (DSS)-induced colorectal carcinogenesis. In Experiment 1, male *Apoe*-deficient (*n* = 20) and wild type (WT) mice (C57BL/6J, *n* = 21) were treated with a single intraperitoneal (i.p.) injection of AOM (10 mg/kg body weight) and then given 1.5% DSS in drinking water for seven days. They were maintained up to week 20 and sacrificed for the histopathological examination of colorectal tumors. The mRNA expression of cyclooxygenase (*Cox*)-2, inducible nitric oxide synthase (*Nos2*), tumor necrosis factor (*Tnf*)-*α* interleukin (*Il*)*-1β*, and *Il-6* was assayed in the colorectal mucosa. In Experiment 2, male *Ldlr*-deficient (*n* = 14) and WT mice (C57BL/6J, *n* = 10) were given a single i.p. injection of AOM (10 mg/kg body weight) and then given 2% DSS in drinking water for seven days. They were sacrificed at week 20 to evaluate their colorectum histopathologically. In Experiment 1, the multiplicity of CRCs was significantly higher in the *Apoe*-deficient mice (2.75 ± 1.48) than in the WT mice (0.62 ± 0.67). The serum lipoprotein levels in the *Apoe*-deficient mice were also significantly higher than in the WT mice. In Experiment 2, the incidence (29%) and multiplicity (0.50 ± 0.94) of CRCs in the *Ldlr* mice were significantly lower than in the WT mice (80% incidence and 3.10 ± 2.38 multiplicity). The mRNA expression of two inducible enzymes and certain pro-inflammatory cytokines in the colorectum of each genotype was greater than in the respective WT mice. The values in the *Apoe*-deficient mice were much greater than in the *Ldlr* mice. These findings suggest that *Apoe*-deficient mice showed increased susceptibility to inflammation-associated colorectal carcinogenesis due to their high reactivity to inflammatory stimuli.

## 1. Introduction

Colorectal cancer (CRC) is the second-most common malignancy worldwide in women and the third-most common malignancy in men [1], although global CRC incidence and mortality have marked variation [1,2]. CRC occurs initially by mutation of the tumor suppressor gene, *APC*, and thereafter via the accumulation of other genetic mutations in a step-wise process over several years [3]. Both hereditary and environmental factors contribute to CRC development [3]. Dietary factors play a particularly important role in colorectal carcinogenesis [3,4,5]. While diets high in red meat and processed meat can increase the risk of CRC, diets rich in fruits, vegetables, and fiber reduce the CRC risk [6,7,8,9]. Animal fat is known to be one of the risk factors for CRC [10,11]. Genetic alterations involving the lipid transportation and its metabolism are also susceptibility factors for CRC [12].

Inflammatory bowel disease (IBD), including Crohn’s disease (CD) and ulcerative colitis (UC), has emerged as a global disease with increasing incidence and prevalence in the world [13,14]. Although the precise etiology of IBD is not known, immune dysregulation induced by genetic and/or environmental factors plays an important role in this complex multifactorial disease [15]. Hyper-inflammation status, such as with chronic hepatitis C or B, reflux esophagitis, and IBD, increases the risk of cancer development in the inflamed tissues [16]. Inflammation also strongly promotes carcinogenesis [17]. Inflammatory mediators, including cytokines, chemokines, reactive oxygen/nitrogen species, prostaglandins, growth and transcription factors, microRNAs, and enzymes (cyclooxygenase and matrix metalloproteinase), collectively establish a microenvironment that is favorable for cancer development through an extensive and dynamic crosstalk with tumor cells. They could cause DNA damage (initiation) and affect the stages of tumor promotion and progression.

Apoe (34KD-MW protein) is the protein product of a single gene mapped to the long arm of chromosome 19q [18,19]. This 50 kilobases (kb) gene cluster includes the genes for apolipoprotein C-I and C-II involved in the regulation of the metabolism of plasma lipoprotein [18,19]. Apoe contains 299 residues and plays an important role in mediating receptor-dependent lipoprotein uptake. The receptors involved in lipoprotein uptake mediated by Apoe are the low-density lipoprotein receptor (Ldlr) and the chylomicron remnant receptor, the latter being also referred to as the Ldlr-related protein (LRP) [20]. The Ldlr is considered to be ubiquitously expressed in mammals, although the bulk of receptor-dependent clearance of LDL is shown to occur through the liver [20,21]. Although there is little information on the distribution of these receptors in human colonic crypt cells, Ldlr is reported to be present in the crypt cells in rat small intestine [22]. In addition, small intestinal crypt cells in rats are able to uptake chylomicron remnants receptor-dependently [23]. 

Apoe may influence CRC development via three potential pathways. They include (1) metabolism of cholesterol and bile acid; (2) regulation of triglycerides (TG) and insulin; and (3) inflammation. Apoe involved in lipid metabolism may affect the absorption of luminal cholesterol and bile acid metabolism [24,25,26]. Possessing an e4 *Apoe* allele may increase the risk of gallstones formation [27,28]. Bile acid is important in CRC development. People with gallstones are at a higher risk of developing proximal colon cancer than those without [29]. Variants of Apoe affect serum levels of lipid and/or triglyceride and insulin sensitivity [30,31]. TG and insulin are known to be involved in CRC development [32,33]. The third pathway via which Apoe regulates CRC development is colonic inflammation [34,35]. Both a high-fat diet and obesity are associated with CRC development [7,36,37].

CRCs contain high levels of fatty acids or their products stored in cancer cell membranes. This suggests a certain role of fatty acids in colorectal carcinogenesis [38,39]. Linoleic acid is converted to arachidonic acid (AA), which is further biosynthesized into prostaglandins (PGs). The *Ldlr* regulates the uptake of essential fatty acid and cholesterol into cells. Then, the essential polyunsaturated fatty acids are esterified to phospholipids. AA released from phospholipids is oxidized by cytochrome P-450 (Cyp), lipoxygenase (Lox), or cyclo-oxygenase (Cox). Through the Cox pathway, various PGs, including PGE_2_ are produced. *Ldlr* plays an important role in the initial uptake of essential fatty acid and the subsequent biosynthesis of eicosanoids, such as PGE_2_ [40,41]. Thus, epidemiological and experimental data suggest fatty acids as an important factor in the CRC development. Over-expression of *Cox-2*, an inducible inflammatory enzyme that metabolizes the essential fatty acids into PGs, in CRC was first observed in 1994 [42], and thereafter the concept of chemoprevention using Cox inhibitors was proposed [42,43,44,45,46]. 

Certain cancers, including CRC and human cancer cell lines, have increased levels in Ldlr protein [47,48]. A loss of feedback regulation of *Ldlr* in CRCs was also reported [49]. In addition, Cox-2 was up-regulated in colorectal neoplasms that over-expressed *Ldlr* mRNA compared with normal colorectal mucosa. These findings may suggest that *Ldlr* is abnormally regulated in tumors and may play a certain role in the up-regulation of *Cox-2* in neoplasms.

Alterations in the plasma lipid profiles and in intracellular cholesterol homoeostasis were reported in various malignancies, including CRCs. However, the significance of these alterations, if any, in colorectal carcinogenesis and cancer biology is not clear. In the current study, we examined whether or not *Apoe* and *Ldlr* were involved in colorectal carcinogenesis in mice. For this, we used an inflammation-associated colorectal carcinogenesis model of *Apoe*- or *Ldlr*-deficient mice developed with azoxymethane (AOM) and promoted by dextran sodium sulfate (DSS), where many colorectal neoplasms develop within a short period of time [50].

## 2. Results

### 2.1. AOM/DSS-Induced Colorectal Carcinogenesis in the Apoe-Deficient Mice (Experiment 1)

**Figure 1:**
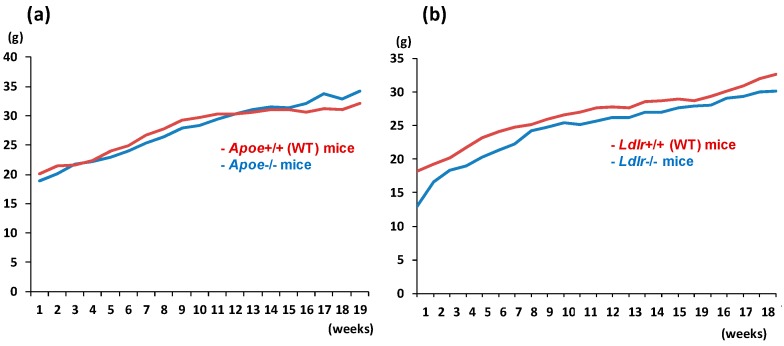
The body weight changes of (**a**) the *Apoe*-deficient and wild type (WT) mice during Experiment 1 and (**b**) the low-density lipoprotein receptor (*Ldlr*)-deficient and WT mice during Experiment 2.

**Figure 2:**
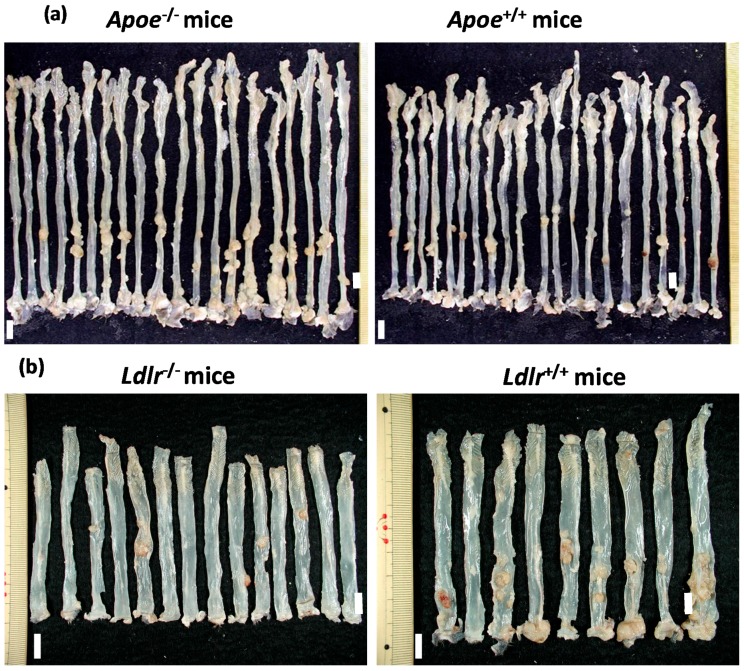
A macroscopic view of the colorectum of (**a**) the *Apoe*-deficient and WT mice and (**b**) the *Ldlr*-deficient and WT mice. Scales, 10 mm.

**Figure 3:**
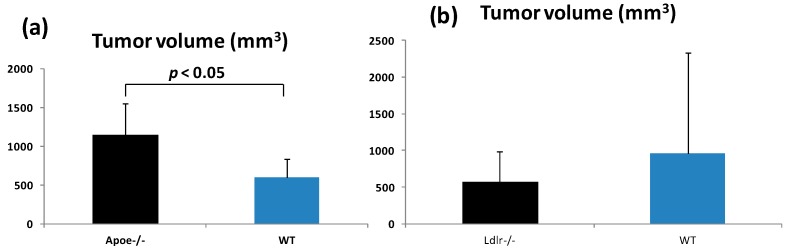
The mean volumes (±SD) of colorectal tumors in (**a**) the *Apoe*-deficient and WT mice and (**b**) the *Ldlr*-deficient and WT mice.

**Figure 4:**
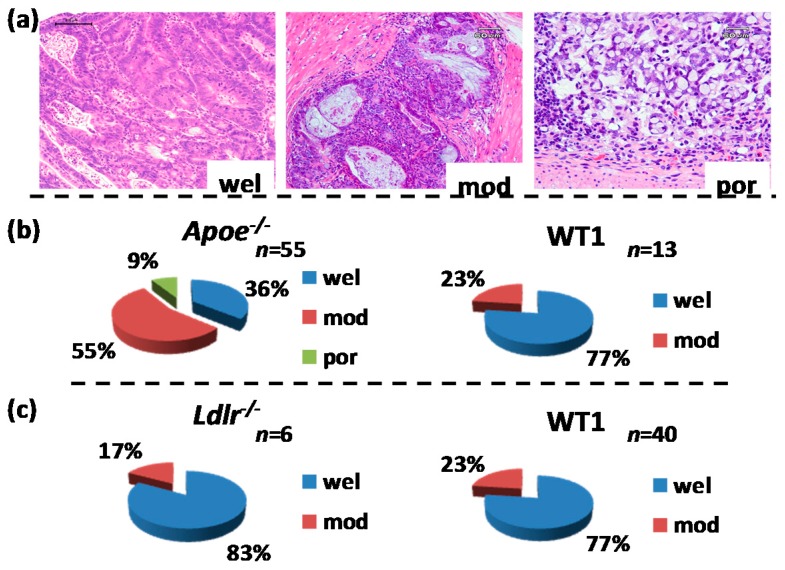
Histopathological analysis of induced colorectal adenocarcinomas. (**a**) Representative histopathology of colonic adenocarcinomas induced by azoxymethane (AOM) and dextran sodium sulfate (DSS). They were classified into three types of differentiation, well-differentiated (wel), moderately differentiated (mod), and poorly differentiated (por). Hematoxylin and eosin (H & E) stain used. Bars are 100 µm in “wel”, 60 µm in “mod”, and 60 µm in “por”; (**b**) percentages of adenocarcinomas developed in the *Apoe*-deficient and wild (C57BL/6J) mice that received AOM and DSS; (**c**) percentage of adenocarcinomas developed in the *Ldlr*-deficient and wild (C57BL/6J) mice that received AOM and DSS.

Both the *Apoe*-deficient mice and WT mice tolerated treatment with AOM and 1.5% DSS well and survived to week 20 (Figure 1a). As listed in Table 1, at sacrifice, the body (*p* < 0.05) and liver weights (*p* < 0.001) of the *Apoe*-deficient mice were significantly greater than those of the WT mice. The mean relative liver weights of both groups were comparable. When given AOM and DSS, the colon length of the *Apoe*-deficient mice was slightly shorter than that of the wild type of mice (Table 1). Treatment with AOM followed by 1.5% DSS resulted in the development of colorectal tumors in both genotypes (Figure 2a). The incidences and multiplicities of several colorectal lesions, such as mucosal ulcer, adenoma (AD), and AD + adenocarcinoma (ADC), were larger in the *Apoe*-deficient mice than in the WT mice, but the differences between the two genotypes were not significant (Table 2). In addition, the multiplicities of dysplastic lesions (DYS) and ADC in the *Apoe*-deficient mice were also larger than in the WT mice, and these differences were statistically significant (dysplastic lesions, *p* < 0.02; and ADC, *p* < 0.005, Table 2). The mean volume (1150.2 ± 396.7 mm^3^) of colorectal tumors in the *Apoe*-deficient mice was significantly greater than that (597.9 ± 234.6 mm^3^) in the WT mice (*p* < 0.05), as shown in Figure 3a. Histopathologically, three types (well, moderately, and poorly) of differentiation were observed in ADCs (Figure 4a). Poorly differentiated adenocarcinomas developed in a few *Apoe*-deficient mice that received AOM and DSS (Figure 4b). The incidence and multiplicity of mucosal ulcer in the *Apoe-*deficient mice were higher than in the WT mice, but the differences were not statistically significant (Table 2).

With regard to the serum biochemistry (Table 3), the cholesterol (*p* < 0.001), very-low-density lipoprotein (VLDL, *p* < 0.001), and low-density lipoprotein (LDL, *p* < 0.001) levels in the *Apoe*-deficient mice were significantly higher than in the WT mice. However, the serum TG, high-density lipoprotein (HDL), glucose, and adiponectin levels were comparable between the genotypes.

**Figure 5:**
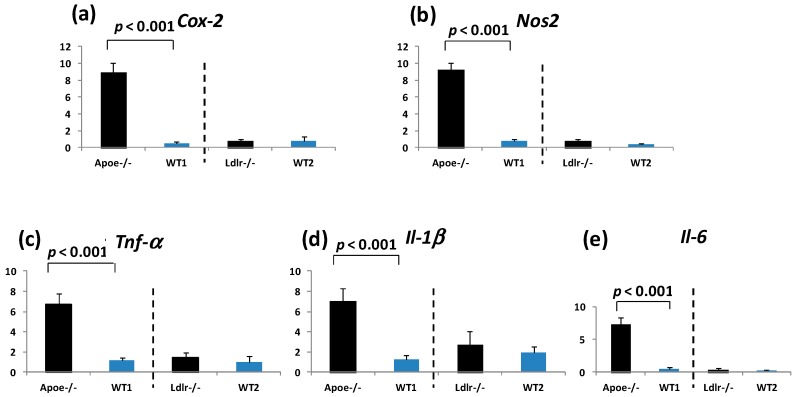
The mRNA expression of (**a**) *Cox-2*; (**b**) *Nos2*; (**c**) *Tnf-α*; (**d**) *Il-1β*; and (**e**) *Il-6* in the colorectal mucosa of the *Apoe*-deficient, *Ldlr*-deficient, and their respective wild mice that received AOM and DSS. The mRNA levels of these molecules were measured by Real-Time Quantitative Polymerase Chain Resction. The expression of all five molecules was significantly higher in the *Apoe*-deficient mice than in the WT mice (*p* < 0.001). The expression of the molecules was slightly but not significantly higher in the *Ldlr*-deficient mice than in the WT mice. The expression was normalized to the β-actin mRNA expression level. The data are represented as the means ± SD from three independent assays (*n* = 5 from each group). *Y*-axis shows expression of the mRNA relative to the “Standard condition” and normalized to β-actin.

The mRNA expression of *Cox-2* (Figure 5a), *Nos2* (Figure 5b), *Tnf-α* (Figure 5c), *Il-1β* (Figure 5d), and *Il-6* (Figure 5e) in the non-lesional colorectal mucosa of the *Apoe*-deficient mice was significantly greater than in the WT mice (*p* < 0.001).

### 2.2. AOM/DSS-Induced Colorectal Carcinogenesis in the Ldlr-Deficient Mice (Experiment 2)

The *Ldlr*-deficient mice and WT mice tolerated treatment with AOM and 2% DSS well and survived to week 20 (Figure 1b). At sacrifice, the body, liver, and relative liver weights of the *Ldlr*-deficient mice and WT mice were almost comparable (Table 1). The colon length of the *Ldlr*-deficient mice was slightly smaller than that of the wild type of mice (Table 1). Treatment with AOM followed by 2% DSS resulted in the development of colorectal tumors in both genotypes (Figure 2b). The incidences (*p* < 0.02 or *p* < 0.05) and multiplicities (*p* < 0.001) of several proliferative colorectal lesions, including DYS, AD, ADC, and AD + ADC, in the *Ldlr*-deficient mice were significantly lower than in the WT mice (Table 2). The mean volume (569.8 ± 417.6 mm^3^) of tumors in the *Ldlr*-deficient mice was smaller than that in the WT mice (966.2 ± 1362.8 mm^3^), but the difference was not statistically significant (Figure 3b). Well- and moderately differentiated ADCs (Figure 4a) developed in the colorectum, but there were no poorly differentiated types (Figure 4c). The incidence and multiplicity of mucosal ulcer in the *Ldlr*-deficient mice were lower than in the WT mice, but the differences were not statistically significant (Table 2). 

The serum levels of cholesterol (*p* < 0.001), TG (*p* < 0.001), LDL (*p* < 0.001), and adiponectin (*p* < 0.01) in the *Ldlr*-deficient mice were significantly higher than in the WT mice (Table 3). However, the serum VLDL, HDL, and glucose levels were comparable between the genotypes.

As illustrated in Figure 5, the mRNA expression of *Cox-2*, *Nos2*, *Tnf-α*, Il-1β, and *Il-6* in the non-lesional colorectal mucosa of the *Ldlr*-deficient mice was slightly elevated when compared to the WT mice.

## 3. Discussion

This is the first study, to our knowledge, to examine the susceptibility of inflammation-associated colorectal carcinogenesis in *Apoe*- and *Ldlr*-deficient male mice in comparison with their background genotype using our AOM/DSS model [50]. Our findings here suggest that *Apoe* and *Ldlr* inversely affect inflammation-associated colorectal carcinogenesis, irrespective of their serum lipoprotein profiles. Surprisingly, the *Apoe*-deficient mice were much more susceptible to AOM/DSS-induced colorectal carcinogenesis than the WT mice. The mRNA expression levels of two inducible enzymes, *Cox-2* and *Nos2*, and three pro-inflammatory cytokines, *Tnf-α*, *Il-1β*, and *Il-6*, in the colorectum of *Apoe*-deficient mice were much higher than in the *Ldlr-*deficient mice.

When given AOM and DSS, the colon length of the *Apoe*-deficient mice or *Ldlr*-deficient mice was shorter than their respective wild mice (Table 1) without statistical significance. This may be related to the findings of severe colitis and enhancement of colorectal carcinogenesis in the *Apoe*-deficient mice or *Ldlr*-deficient mice. Histopathologic investigation revealed that poorly differentiated ADCs were developed only in the *Apoe*-deficient mice that received AOM and DSS, but not in the *Ldlr*-deficient mice and their respective wild mice (Figure 4b,c). Severe inflammation and increased levels of Cox-2, Nos2, Tnf-α, Il-1β, and Il-6 observed in the colorectum of *Apoe*-deficient mice treated with AOM and DSS may be related to these differences of histopathological findings of ADCs.

The *Apoe*- and *Ldlr*-deficient mice, which have elevated serum levels of cholesterol, TG, VLDL, LDL, and/or HDL, are frequently used for research of atherosclerosis and in developing new drugs against atherosclerosis [51,52]. In contrast to the *Ldlr*-deficient mice, the *Apoe*-deficient mice develop atherosclerosis spontaneously without an atherogenic diet. The lipid profiles of these mice differ slightly. For example, hypercholesterolemia in the *Apoe*-deficient mice is more severe than in the *Ldlr*-deficient mice. Compared to the WT mice, the serum VLDL level of the *Apoe*-deficient mice is markedly increased (five-fold that of the WT mice [51]), but the elevation in the VLDL level of the *Ldlr*-deficient mice is moderate. While the serum HDL levels of the *Apoe*-deficient mice are decreased (45% of that in the WT mice [51]), the levels in the *Ldlr*-deficient mice are modestly increased. The TG level in the *Apoe*-deficient mice was found to be 68% higher than that in the WT mice [51]. The different susceptibilities of the two genotypes to AOM/DSS-induced colorectal carcinogenesis may be due to their differing lipid profiles. However, the precise reason for the differences in susceptibility observed in this study is unclear.

*Apoe* is a major modulator of lipoprotein metabolism, and the allele-specific effects of *Apoe* on lipoprotein metabolism were reported [53]. *Apoe* also has other crucial functions, and aberration of these functions may lead to carcinogenesis [54,55,56,57]. In vitro studies suggest that treatment of the colon cancer cell line, HT29, with *Apoe* increased the cell polarity by translocating β-catenin from the cytoplasm to cell–cell adhesion sites [56]. *Apoe* is able to inhibit cell proliferation and de novo DNA biosynthesis [57]. Functional experiments on *Apoe* isoforms showed that *Apoe4*, but not wild-type *Apoe*, inhibits glycogen synthase kinase (GSK)-3β and increases the amount of active protein kinase B (PKB), which further inactivates GSK-3β, leading to enhancement of β-catenin translocation into nuclei [54]. Nuclear β-catenin can promote transcription of genes involved in cell survival and division [58,59,60]. Since patients with UC show impaired lipoprotein metabolism [61], the effect of *Apoe* polymorphism on the risk of UC development should be investigated. 

In the present study, we assayed the mRNA expression of *Cox-2*, *Nos2*, *TNF-α*, *Il-1β*, and *Il-6* in the colorectal mucosa of the *Apoe*-deficient, *Ldlr*-deficient, and their respective WT mice. The values of the *Apoe*-deficient mice treated with AOM and DSS were significantly and much greater than the WT mice that received AOM and DSS, suggesting that the *Apoe*-deficient mice have hyper-inflammation status. Sensitivity to inflammatory stimuli was reported to be greater in *Apoe*-deficient mice [62] than in *Ldlr*-deficient mice [63]. This was related to a decrease in the production of certain pro-inflammatory cytokines in the *Ldlr*-deficient mice, although macrophages maintained an elevated cytokine production capacity [62,63]. *Apoe* involved in cholesterol and lipid metabolism has also altered both innate and adaptive immune responses [64]. Importantly, mice lacking *Apoe* exhibit increased inflammatory responses and higher mortality following lipopolysaccharide challenge [65]. This may suggest that *Apoe* has anti-inflammatory effects. Genetic factors have been reported to contribute to the pathophysiology of IBD [66]. *Apoe* inhibits the production of T lymphocytes and regulates immune reactions by interacting with several cytokines [67,68]. *Apoe* thus plays a key role in regulating the immune response in various autoimmune diseases [69].

Pro-inflammatory cytokines are central mediators of the chronic inflammatory process in several tissues. IL-6 is part of a central pathway in the pathogenesis of chronic inflammation diseases, such as IBD [70], and inflammation-associated colorectal cancer [71,72,73]. *IL-6* trans-signaling is also an independent risk factor for coronary artery disease and is involved in inflammatory processes in vessels [74]. Other cytokines (*Tnf-α*, *IL-1β*, *Interferon-γ*), inflammatory enzymes (*Cox-2*, *Nos2*), nuclear factor (NF)-κB, and signal transducer and activator of transcription 3 (Stat3) are also involved in inflammation-associated colorectal carcinogenesis [72]. Uncontrolled activation of Nf-κB, Stat3, and Wnt-β-catenin signaling pathways enhances the aberrant proliferation of crypal cells in the sustained inflammatory microenvironment and promotes CRC development [75]. Although we did not assay Stat3 and Nf-κB in the present study, the mRNA expression of *Tnf-α*, *Il-1β*, *Il-6*, *Cox-2*, and *Nos2* was assayed in the colorectum of *Apoe*-deficient, *Ldlr*-deficient, and their respective WT mice treated with AOM and DSS. The expression of all molecules was greater in the *Apoe*-deficient mice than that of the WT mice. The expression of *Nos2*, *Tnf-α*, and *Il-1β* in the Ldlr-deficient mice was slightly higher than that of the WT mice. The *Apoe*-deficient mice showed even more elevation of these molecules than the *Ldlr*-deficient mice. Thus, the *Apoe*-deficient mice were in a higher-inflammation status compared to the *Ldlr*-deficient mice. Further studies should investigate colorectal carcinogenesis in *Apoe*/*Ldlr* double-knockout mice.

## 4. Materials and Methods

### 4.1. Animals, Chemicals, and Diets

Twenty male five-week-old *Apoe*^−/−^ mice (C57BL/6J background, Jackson Laboratories, Bar Harbor, ME, USA), 14 male five-week-old *Ldlr*^−/−^ mice (C57BL/6J background, Jackson Laboratories), and 31 male C57BL/6J mice were used in this study. Two experiments were conducted. The experiment for AOM/DSS-induced colorectal carcinogenesis in the *Apoe*-deficient mice (Experiment 1) was conducted using *Apoe*^−/−^ (*n* = 20) and C57BL/6J mice (*n* = 21), and contained two experimental groups that were treated with AOM and 1.5% DSS in their drinking water. Similarly, the experiment for AOM/DSS-induced colorectal carcinogenesis in the *Ldlr*-deficient mice (Experiment 2) was conducted using *Ldlr*^−/−^ (*n* = 14) and C57BL/6J mice (*n* = 10), and contained two experimental groups that were given AOM and 2% DSS in their drinking water. A colorectal carcinogen AOM was obtained from Sigma-Aldrich Chemical (St. Louis, MO, USA), and DSS with a molecular weight of 36,000–50,000 Da (Lot No. 6046H) was purchased from MP Biomedicals (Aurora, OH, USA). AOM was diluted in physiological saline just before injection. DSS for colitis induction was dissolved in water at 1.5% (*w*/*v*) or 2% (*w*/*v*). A pelleted Charles River Formula (CRF)-1 diet (Oriental Yeast, Tokyo, Japan) was used as the basal diet throughout the study. All of the mice were maintained in the animal facility of the University, according to the Institutional Animal Care Guidelines. The animals were housed in plastic cages (three to five mice/cage) with free access to tap water and CRF-1 (Oriental Yeast Co., Ltd., Tokyo, Japan), under controlled conditions of humidity (50% ± 10%), light (12/12 h high/dark cycle), and temperature (23 ± 2 °C). Study designs were approved by the University and animal handling and procedures were performed in accordance with the Institutional Animal Care Guidelines.

### 4.2. Study Design

**Figure 6:**
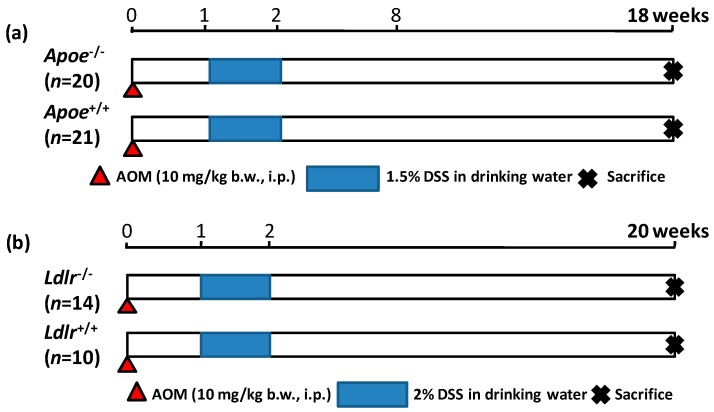
The experimental protocol of (**a**) the *Apoe*-deficient and WT mice that received AOM and DSS (Experiment 1) and (**b**) the *Ldlr*-deficient and WT mice that received AOM and DSS (Experiment 2). i.p., intraperitoneal.

In the AOM/DSS-induced colorectal carcinogenesis in the *Apoe*-deficient mice (Experiment 1), twenty five-week old *Apoe*-deficient male mice and twenty-one, five-week old C57BL/6J male mice were given a single intraperitoneal (i.p.) injection of AOM (10 mg/kg body weight), as shown in Figure 6a. One week after the injection, they received 1.5% DSS in their drinking water for seven days and then were maintained on a basal diet and tap water for 18 weeks. In the AOM/DSS-induced colorectal carcinogenesis in the *Ldlr*-deficient mice (Experiment 2), fourteen five-week-old *Ldlr*-deficient male mice and ten five-week-old C57BL/6J male mice were given a single i.p. injection of AOM (10 mg/kg body weight), as shown in Figure 6b. Similar to Experiment 1, they received 2% DSS in their drinking water for seven days and then were maintained on a basal diet and tap water for 18 weeks. In both experiments, all of the animals were killed by an overdose of ether at week 20 to evaluate the colorectal lesions histopathologically. 

At sacrifice, complete necropsy was done on all mice. The body and liver weights were measured and processed for a histopathological examination by conventional methods. The colon was flushed with normal saline and then removed. After measuring the length, the colon was cut open longitudinally along the main axis and gently washed with normal saline. The colonrectum was macroscopically inspected for the presence of lesions and fixed in 10% buffered formalin for at least 24 h. A histopathological examination was conducted on paraffin-embedded sections after hematoxylin and eosin (H & E) staining. 

### 4.3. Real-Time Quantitative Polymerase Chain Reaction (RT-PCR)

We determined the mRNA expression in the non-lesional colorectal mucosa from five mice of each genotype, *Apoe*-deficient mice, *Ldlr*-deficient mice, or their respective wild type of mice.

RNA was extracted from the olorectum and stored at −80 °C using TRIzol reagent (Thermo Fisher Scientific, K.K., Yokohama, Japan) according to the manufacturer’s protocol. RNA concentration and quality were verified, and reversely transcribed to produce cDNA. Quantitative RT-PCR analyses of *Cox-2*, *Nos2*, *Tnf-α*, *Il-1β*, and *Il-6* were performed with ABI Prism 7500 (Applied Biosystems Japan Ltd., Tokyo, Japan) using TaqMan Gene Expression Assays (Applied Biosystems Japan Ltd., Tokyo, Japan): *Cox-2* (*Ptgs2*), Mm00478374-ml; *Nos2* (Mm00440485-ml); *Tnf-α*, Mm00443258-m1; *Il-1β*, Mm00434228**-**m1; and *IL-6*, Mm00446190-ml. *β-Actin* (Mm00607939-sl) was used to normalize the expression level of the mRNA genes. The cycling protocol of RT-PCR was conducted at a DNA denaturation temperature of 95 °C for 5 min and followed by 40 cycles of 95 °C for 15 s, 60 °C for 20 s, and an elongation temperature 72 °C for 40 s. Each experiment was performed in triplicate, and data were calculated by ΔΔ*C*_t_ methods.

### 4.4. Clinical Chemistry (Serum Lipid Profiles)

At sacrifice, blood samples were collected to measure the serum concentrations of total cholesterol, TG, very-low-density lipoprotein (VLDL), LDL, high-density lipoprotein (HDL), glucose, and adiponectin after overnight fasting from 10 or 14 mice in each group. Whole blood anti-coagulated with heparin lithium was taken from the inferior vena cava with a sterile syringe (Terumo, Tokyo, Japan). The serum was obtained by centrifugation (3000 rpm for 10 min), and stored at −80 °C until measurement. The serum TG and total cholesterol levels were determined using commercial enzymatic assay kits (TG, L-Type WAKO-TG·H; and total cholesterol, L-Type WAKO-CHO·H), obtained from Wako Pure Chemical Industries, Ltd. (Osaka, Japan). The serum levels of HDL, LDL, and VLDL were determined using an HDL and LDL/VLDL Cholesterol Quantitation Kit (BioVision, Inc., Milpitas, CA, USA). The serum glucose level was determined using commercial enzymatic assay kit (Glucose CII-test WAKO, Wako Pure Chemical Industries). These measurements were expressed as mg/dL. The serum adiponectin level (µg/mL) was determined with Mouse/Rat Adiponectin ELISA kits (Otsuka Pharmaceutical Co., Ltd., Tokyo, Japan).

### 4.5. Statistical Analysis

The incidences of colonic lesions between the groups were compared using the chi-square test or Fisher’s exact probability test (GraphPad Instat version 3.05; GraphPad Software, San Diego, CA, USA). Other measures expressing mean ± standard deviation (SD) were statistically analyzed using one-way analysis of variance (ANOVA), followed by the Bonferroni or Tukey-Kramer multiple comparison post-test (GraphPad Instat version 3.05; GraphPad Software). mRNA expression was statistically analyzed by the Kruskal-Wallis test. Differences were considered statistically significant at *p* < 0.05.

## 5. Conclusions

Our findings indicate that *Apoe* and *Ldlr* are inversely involved in inflammation-associated colorectal carcinogenesis induced by AOM/DSS, irrespective of their serum lipoprotein profiles: the *Apoe*-deficient mice were much more susceptible to inflammation-associated colorectal carcinogenesis than the WT mice. The mRNA expression levels of two inducible enzymes and certain pro-inflammatory cytokines in the colorectum of *Apoe*-deficient mice were much more elevated than in the *Ldlr-*deficient mice.

## Figures and Tables

**Table 1:** Body weight, liver weights, and colon length.

Measurements	*Apoe*-Deficient Mice (*n* = 20)	WT Mice (*n* = 21)	*Ldlr*-Deficient Mice (*n* = 14)	WT Mice (*n* = 10)
Body weight (g)	34.1 ± 2.2 ^a,b^	31.4 ± 3.5	30.1 ± 3.1 ^b^	32.6 ± 1.7
Liver weight (g)	1.31 ± 0.07 ^c^	1.14 ± 0.09	1.43 ± 0.13 ^d^	1.60 ± 0.22
% Liver weight (Liver weight/100 g body weight)	3.77 ± 0.33	3.69 ± 0.36	4.78 ± 0.28	4.91 ± 0.52
Colon length (cm)	12.35 ± 0.90	12.50 ± 0.90	10.09 ± 0.62	10.4 ± 0.72

WT, wild type. ^a^ Mean ± standard deviation (SD); ^b−d^ Significantly different from the respective WT mice (^b^ *p* < 0.05, ^c^ *p* < 0.001, and ^d^ *p* < 0.01).

**Table 2:** Multiplicity and incidence of colorectal preneoplasia and neoplasia.

Pathological Lesions	*Apoe*-Deficient Mice (*n* = 20)	WT Mice (*n* = 21)	*Ldlr*-Deficient Mice (*n* = 14)	WT Mice (*n* = 10)
Mucosal ulcer	1.25 ± 1.74 ^a^	0.57 ± 0.98	2.71 ± 2.13	3.40 ± 1.65
	(10/20, 50%)	(7/21, 33%)	(12/14, 86%)	(10/10, 100%)
Dysplastic crypts	1.05 ± 1.50	0.14 ± 0.48	0.43 ± 0.65 ^c^	4.30 ± 2.67
	(9/20, 45% ^b^)	(2/21, 10%)	(5/14, 36% ^b^)	(9/10, 90%)
Adenoma (AD)	1.00 ± 1.08	0.62 ± 0.74	0.29 ± 0.61 ^c^	2.10 ± 1.52
	(11/20, 55%)	(10/21, 48%)	(3/14, 21% ^b^)	(8/10, 80%)
Adenocarcinoma (ADC)	2.75 ± 1.48 ^c^	0.62 ± 0.67	0.50 ± 0.94 ^c^	3.10 ± 2.38
	(19/20, 95% ^d^)	(11/21, 52%)	(4/14, 29% ^e^)	(8/10, 80%)
AD + ADC	3.75 ± −1.83 ^c^	1.24 ± 0.94	0.79 ± 1.31 ^c^	5.20 ± 3.12
	(20/20, 100%)	(17/21, 81%)	(5/14, 36% ^e^)	(8/10, 80%)

WT, wild type. ^a^ Mean ± standard deviation (SD); ^b−e^ Significantly different from the respective WT mice (^b^ *p* < 0.02, ^c^ *p* < 0.001, ^d^ *p* < 0.005, and ^e^ *p* < 0.05).

**Table 3:** Serum lipoprotein profiles.

Measurements	*Apoe*-Deficient Mice (*n* = 21)	WT Mice (*n* = 20)	*Ldlr*-Deficient Mice (*n* = 14)	WT Mice (*n* = 10)
Serum cholesterol (mg/dL)	628 ± 110 ^a,b^	132 ± 23	414 ± 41 ^b^	131 ± 21
Serum triglycerides (mg/dL)	106 ± 16	85 ± 28	230 ± 51 ^b^	126 ± 21
Serum VLDL (mg/dL)	403 ± 99 ^b^	110 ± 17	149 ± 4	120 ± 4
Serum LDL (mg/dL)	439 ± 170 ^b^	49 ± 12	287 ± 11 ^b^	42 ± 5
Serum HDL (mg/dL)	36 ± 32	59 ± 16	81 ± 3	63 ± 35
Serum glucose (mg/dL)	147 ± 15	175 ± 25	181 ± 39	184 ± 14
Serum adiponectin (µg/mL)	12.9 ± 2.3	12.0 ± 1.7	16.6 ± 2.1 ^b^	12.1 ± 3.5

HDL, high-density lipoprotein; LDL, low-density lipoprotein; VLDL, very-low-density lipoprotein; WT, wild type. ^a^ Mean ± standard deviation (SD); ^b^ Significantly different from the respective WT mice (*p* < 0.001).
